# SARS-CoV-2 Spike N-Terminal Domain Engages 9-*O*-Acetylated α2–8-Linked Sialic Acids

**DOI:** 10.1021/acschembio.3c00066

**Published:** 2023-04-27

**Authors:** Ilhan Tomris, Luca Unione, Linh Nguyen, Pouya Zaree, Kim M. Bouwman, Lin Liu, Zeshi Li, Jelle A. Fok, María Ríos Carrasco, Roosmarijn van der Woude, Anne L. M. Kimpel, Mirte W. Linthorst, Sinan E. Kilavuzoglu, Enrico C. J. M. Verpalen, Tom G. Caniels, Rogier W. Sanders, Balthasar A. Heesters, Roland J. Pieters, Jesús Jiménez-Barbero, John S. Klassen, Geert-Jan Boons, Robert P. de Vries

**Affiliations:** † Department of Chemical Biology & Drug Discovery, Utrecht Institute for Pharmaceutical Sciences, Utrecht University, 3584 CG Utrecht, The Netherlands; ‡ CICbioGUNE, Basque Research & Technology Alliance (BRTA), Bizkaia Technology Park, Building 800, 48160 Derio, Bizkaia, Spain; § Ikerbasque, Basque Foundation for Science, Maria Diaz de Haro 3, 48013 Bilbao, Bizkaia, Spain; ∥ Department of Chemistry, University of Alberta, 11227 Saskatchewan Drive, Edmonton T6G 2G2, Canada; ⊥ Complex Carbohydrate Research Center, University of Georgia, 315 Riverbend Road, Athens, Georgia 30602, United States; # Department of Medical Microbiology, Amsterdam UMC, University of Amsterdam, 1081 HZ Amsterdam, The Netherlands; ∇ Amsterdam Institute for Infection and Immunity, Infectious Diseases, 1081 HZ Amsterdam, The Netherlands; ○ Department of Microbiology and Immunology, Weill Medical Center of Cornell University, 1300 York Avenue, New York, New York 10065, United States; ◆ Department of Organic Chemistry, II Faculty of Science and Technology University of the Basque Country, EHU-UPV, 48940 Leioa, Spain; ¶ Centro de Investigación Biomédica En Red de Enfermedades Respiratorias, Av. Monforte de Lemos, 3-5. Pabellón 11. Planta 0, 28029 Madrid, Spain

## Abstract

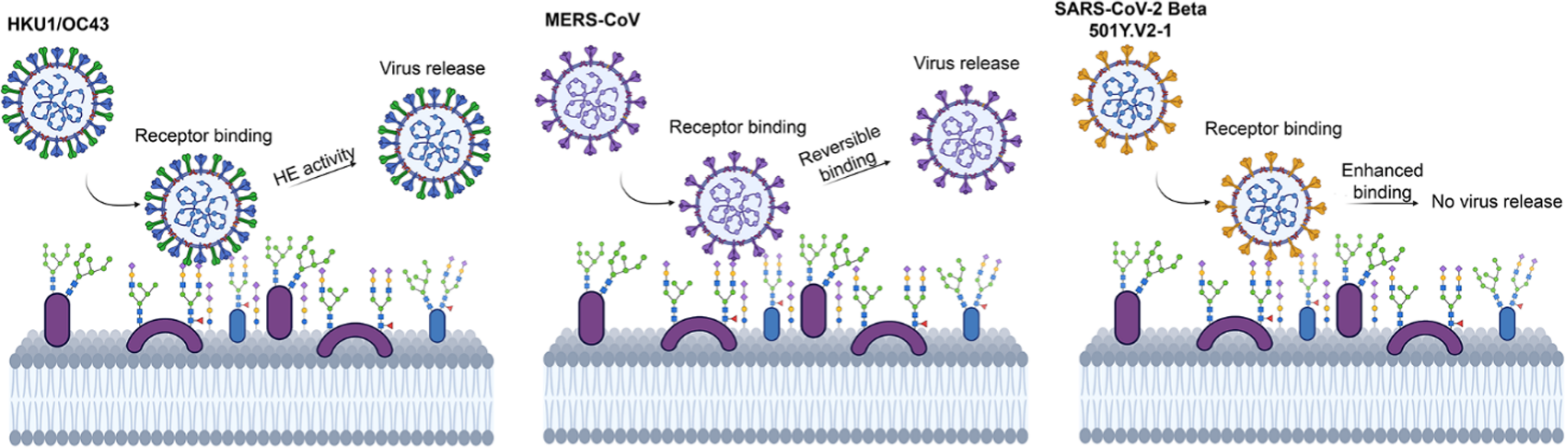

SARS-CoV-2 viruses engage ACE2 as a functional receptor with their spike protein. The S1 domain of the spike protein contains a C-terminal receptor binding domain (RBD) and an N-terminal domain (NTD). The NTD of other coronaviruses includes a glycan binding cleft. However, for the SARS-CoV-2 NTD, protein–glycan binding was only observed weakly for sialic acids with highly sensitive methods. Amino acid changes in the NTD of variants of concern (VoC) show antigenic pressure, which can be an indication of NTD-mediated receptor binding. Trimeric NTD proteins of SARS-CoV-2, alpha, beta, delta, and omicron did not reveal a receptor binding capability. Unexpectedly, the SARS-CoV-2 beta subvariant strain (501Y.V2-1) NTD binding to Vero E6 cells was sensitive to sialidase pretreatment. Glycan microarray analyses identified a putative 9-*O*-acetylated sialic acid as a ligand, which was confirmed by catch-and-release ESI-MS, STD-NMR analyses, and a graphene-based electrochemical sensor. The beta (501Y.V2-1) variant attained an enhanced glycan binding modality in the NTD with specificity toward 9-*O*-acetylated structures, suggesting a dual-receptor functionality of the SARS-CoV-2 S1 domain, which was quickly selected against. These results indicate that SARS-CoV-2 can probe additional evolutionary space, allowing binding to glycan receptors on the surface of target cells.

## Introduction

ACE2 is widely recognized as the functional and essential receptor of SARS-CoV-2.^1,2^ Several reports also demonstrate the importance of the glycocalyx, a thick layer of glycans covering every eukaryotic cell and a known barrier for a plethora of pathogens.^3^ Within this glycocalyx, heparan sulfate moieties have been shown to be an important attachment factor for a variety of viruses including coronaviruses.^4,5^ Another element of the glycocalyx, sialylated glycans, are like heparan sulfates negatively charged and are essential receptors for a wide variety of viruses, again including members of the coronaviruses.^6−8^

Interaction of the SARS-CoV-2 spike glycoprotein toward (sialo)glycans has been confirmed by different studies, albeit without further identification of which domain facilitates this interaction.^9−11^ The spike protein of SARS-CoV-2 consists of several domains that contain a variety of functions. The S2 domain of the spike protein contains the viral fusion machinery. The S1 domain is divided into an N-terminal domain (NTD) and a C-terminal domain, which is referred to as receptor binding domain (RBD) and contains the receptor binding site.^12,13^ The RBD gathered the most attention as it is prone to immune recognition to inhibit receptor binding and thus infection. Continuous immune pressure resulted in the emergence of mutants escaping from neutralizing antibodies and/or with increased ACE2 binding affinity.^14−21^ For the RBD, a (sialo)glycan-dependent attachment mechanism is described, whereby sialic acid and heparan sulfate moieties function as an initial point of attachment.^5,8^ Recently, the NTD of SARS-CoV-2 has been shown to contain a sialic acid binding site,^22,23^ and low-affinity binding has been demonstrated by using saturation transfer difference (STD)-NMR methods. The NTD is also an important antigenic site,^24,25^ indicating its critical role in the viral life cycle with an apparent function.

Several coronaviruses harbor a glycan binding function in the NTD, and using this domain, coronaviruses can bind a wide variety of glycan structures from nonsialylated *N*-glycans to heavily modified sialic acids on glycolipids (Table S1).^26−28^ Many viral pathogens recognize sialic acid as a primary receptor for infection, which occurs in many modified forms and is attached to the underlying glycan structure using certain linkages (α2–3, α2–6, and α2–8).^29^ Even though sialic acids exist in many diverse forms, synthesis is tightly regulated and varies depending on the tissue, physiological conditions, and animal species. Sialic acid consists of nine carbons, and most modifications, such as acetyl, sulfate, lactolyl, and methyl, are commonly present on the 4, 5, 7, 8, and 9 positions.^30,31^ The *O*-acetyl modification is introduced to sialic acids in the Golgi apparatus by sialic acid-specific *O*-acetyltransferases (SOATs) on C-4/7/8/9 and removed by *O*-acetylesterases (SIAEs).^32^ Currently, only a single mammalian SOAT has been identified (CASD1) that resides in the Golgi and transfers the *O*-acetyl to CMP-sialic acid substrate prior to sialyltransferase activity.^33^ The exact functionality of *O*-acetylation is not well defined; however, it appears that *O*-acetylation plays a role in disease and is essential during development, differentiation, and immunological processes.^34^ In addition to (intra-)cellular processes, viral pathogens have evolved to recognize and utilize different *O*-acetylated sialylated structures as receptors to mediate infection.^29^ 9-*O*-Acetylated sialylated structures are variably displayed on cultured human lung cells (A549) and were also found in the human trachea (submucosal glands) and lung (alveolar pneumocytes), as shown with the porcine torovirus hemagglutinin esterase (PToV-HE) virolectin.^30,35^ In addition, 9-*O*-acetylation is also found in different tissues with varying abundance levels.

We wanted to examine whether antigenic drift in the NTD could result in improved sialic acid binding properties. We, therefore, created trimeric NTD of VoC spike proteins and analyzed their binding to Vero E6 cells and tissue slides from several species. Three prevalent variants of 501Y.V2 (beta, B.1.351) were circulating in South Africa originating from SARS-CoV-2 Wuhan with the D614G mutation, lineages 501Y.V2-1, 501Y.V2-2, and 501Y.V2-3.^36^ The early 501Y.V2-1 beta subvariant gained a receptor binding function using its NTD, which appeared to be sialic acid-dependent since sialidase treatment abrogated binding. This NTD–sialoglycan binding functionality was lost in the subvariant 501Y.V2-3, which is commonly referred to as B.1.351 and quickly became the dominant beta variant.^31^

Using glycan arrays, ESI-MS, STD-NMR methods, and a graphene-based sensor, we here demonstrate that the SARS beta subvariant 501Y.V2-1 NTD protein can engage 9-*O*-acetylated α2–8-linked disialic acids in a similar manner to HKU1 NTD. In previous studies, the use of HKU1 and OC43 NTD has been omitted;^11,23^ here, we show the relatively weaker responsiveness of 501Y.V2-1 NTD in comparison to HKU1 and OC43 NTD. This weaker interaction is possibly related to the lack of hemagglutinin esterase activity, thus requiring reversible binding toward target structures. Furthermore, the presence of 9-*O*-acetylated sialylated structures in the trachea and lung indicates that these structures could be an initial binding point for SARS-CoV-2.

## Results

### Beta NTD Protein Gain-of-Function Is Sialic Acid-Dependent

**Figure 1:**
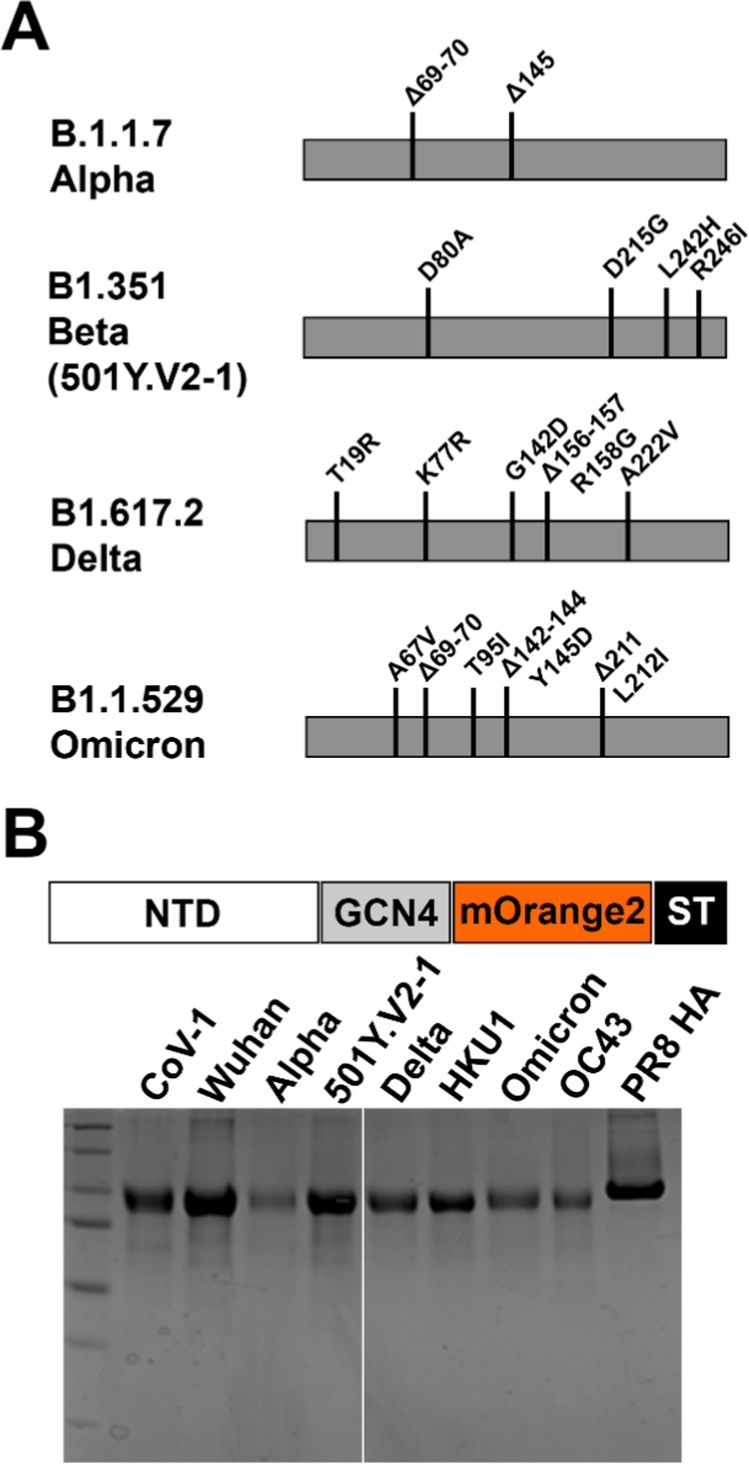
(A) Mutations in VoCs in relation to SARS-CoV-2 Wuhan. (B) NTDs expressed as trimeric proteins using a GCN4 trimerization domain, C-terminally fused to mOrange2 shown on coomassie gel.

**Figure 2:**
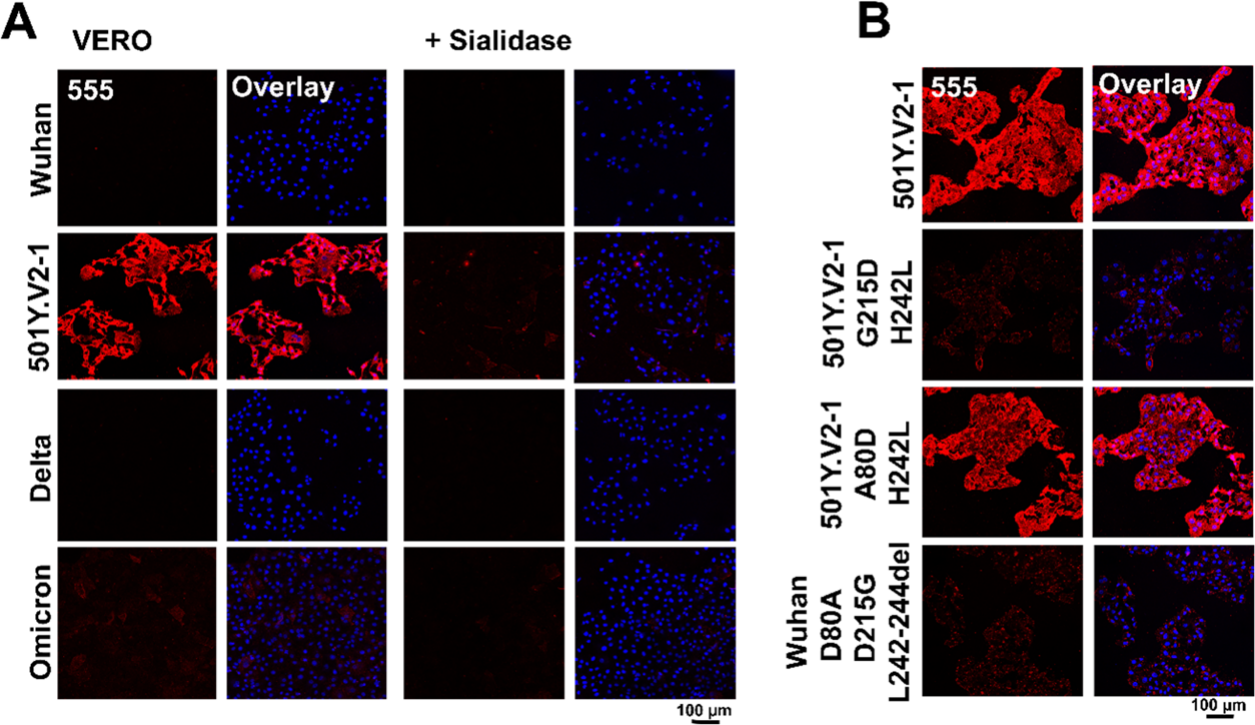
(A) SARS-CoV-2 VoC beta (501Y.V2-1) trimeric NTD protein gaining sialic acid-dependent cell binding, beta NTD receptor binding abrogated with sialidase treatment, no binding observed for SARS-CoV-2 Wuhan, delta, and omicron. (B) Mutations in 501Y.V2-1 NTD (G215D+H242L and A80D+H242L) and in SARS-CoV-2 (D80A+D215G+L242–244del) delineate the importance of amino acid G215D and the amino acids at positions 242–244 being important for receptor binding.

**Figure 3:**
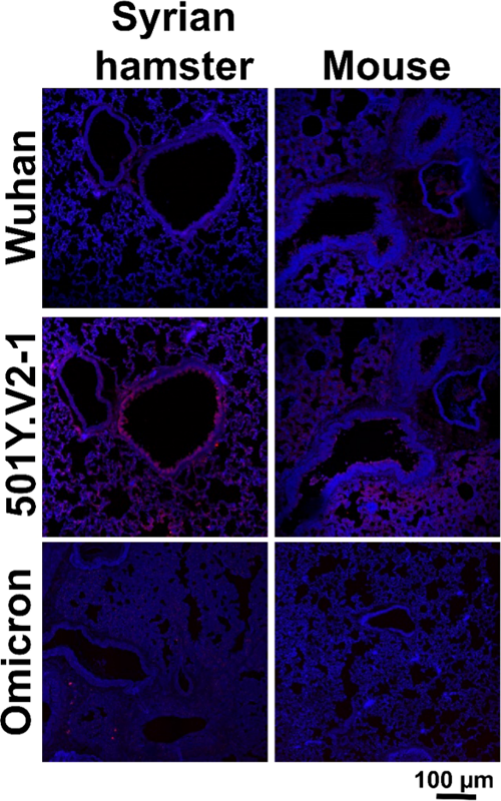
Confocal imaging of SARS-CoV-2, 501.V2-1 and omicron NTDs Syrian hamster and mouse tissue slides. Binding of 501.V2-1 NTD was observed for Syrian hamster and mouse. For Wuhan, minimal fluorescence signal was observed; explicit binding could not be verified.

We started to test recombinant NTD proteins of different VoCs as several other members of the coronavirus family employ this protein domain to bind sialylated or nonsialylated glycans for attachment.^7,28,37−39^ We have previously used trimeric NTDs in our studies to assess glycan binding properties of avian γ-CoVs and have shown that these recapitulate glycan binding of S1 and full ectodomains.^40,41^ To fully rule out the contribution of the RBD in the S1 CTD, we therefore utilized these constructs. The presence of a galectin fold in this domain is known, and previous studies utilizing STD-NMR techniques have reported the binding of SARS-CoV-2 NTD to sialic acid.^22,23,42^ Nevertheless, our attempts to detect binding of SARS-CoV-2 Wuhan NTD to cells and lung tissues, using fluorescent NTD trimers of the S1 spike domain, were not successful as previously described.^43^ Circulation of SARS-CoV-2 and continuous adaptation have led to the emergence of VoCs (alpha, beta, delta, and omicron), with the more recent omicron variant having an unusual number of mutational changes in the NTD (Figure 1A). In particular, the NTDs were expressed with a trimerization domain and a C-terminal fluorescent mOrange2 reporter (Figure 1B). Receptor binding was characterized on Vero E6 cells, commonly used to isolate/propagate SARS-CoV-2 viruses.^44^ For SARS-CoV-2 Wuhan, alpha, delta, and omicron NTD, no tangible signal was detected on Vero E6 cells by confocal imaging (Figures 2 and S1). On the other hand, the 501Y.V2-1 variant did bind efficiently to cells. This binding property appeared to be dependent on the presence of sialic acids since enzymatic treatment with sialidase and 3FNeu5Ac-dependent sialic acid depletion abrogated cell binding (Figures 2A, S1, and S2). Binding of 501Y.V2-1 NTD was further compared to SARS-CoV-2 Wuhan and other VoCs on formalin-fixed, paraffin-embedded lung tissue slides from Syrian hamster and mouse (Figure 3). Similar to cell staining, efficient binding was observed using the 501Y.V2-1 NTD on the lung tissue slides of Syrian hamster and mouse.

The beta variant employed in these experiments retains the amino acids at positions 242–244 with H242L (Figure 1A); this nondominant variant (501Y.V2-1) appeared to circulate initially in South Africa. Five amino acid mutations were identified in the 501Y.V2-1 variant: D80A, D215G, E484K, N501Y, and A701V. Subsequent mutations were introduced into the 501Y.V2-1 variant S protein (L18F and K417N), which resulted in the emergence of 501Y.V2-2. Hereafter, a deletion at 242–244 caused the dominant final variant to appear (501Y.V2-3).^45,46^ The deletions at 242–244 result in the disruption of neutralizing monoclonal antibody binding, facilitating immune escape.^47,48^ These amino acids precede the N5-loop supersite (amino acids 246–260) to which potent antibodies are elicited. Additionally, amino acids at positions 242–244 appeared to be essential in stabilizing the cryptic SARS-CoV-2 NTD binding pocket and facilitate sialic acid interaction.^23^

Further characterization was performed by generating mutants with different combinations in 501Y.V2-1 (G215D+H242L and A80D+H242L) and in SARS-CoV-2 (D80A+D215G+L242–244del) NTD to assess which amino acid mutation/deletion is essential for sialic acid-dependent binding. Cell staining with 501Y.V2-1 G215D+H242L NTD resulted in a loss of cell binding, while staining with 501Y.V2-1 A80D+H242L NTD resulted in a similar receptor binding capacity, as observed using 501Y.V2-1 NTD (Figure 2B). For SARS-CoV-2 Wuhan NTD with mutations D80A, D215G, and L242–244del, no cell binding could be detected. Signal intensity of omicron could not be verified quantitatively; hence, quantification data was not included. Taken together, this data indicates that amino acid mutation D215G is essential and that deletion of amino acids at positions 242–244 is detrimental for sialic acid binding.

### SARS-CoV-2 501Y.V2-1 Variant of Concern Exhibits an Analogous Binding Specificity as Other β-Coronavirus N-Terminal Domains

**Figure 4:**
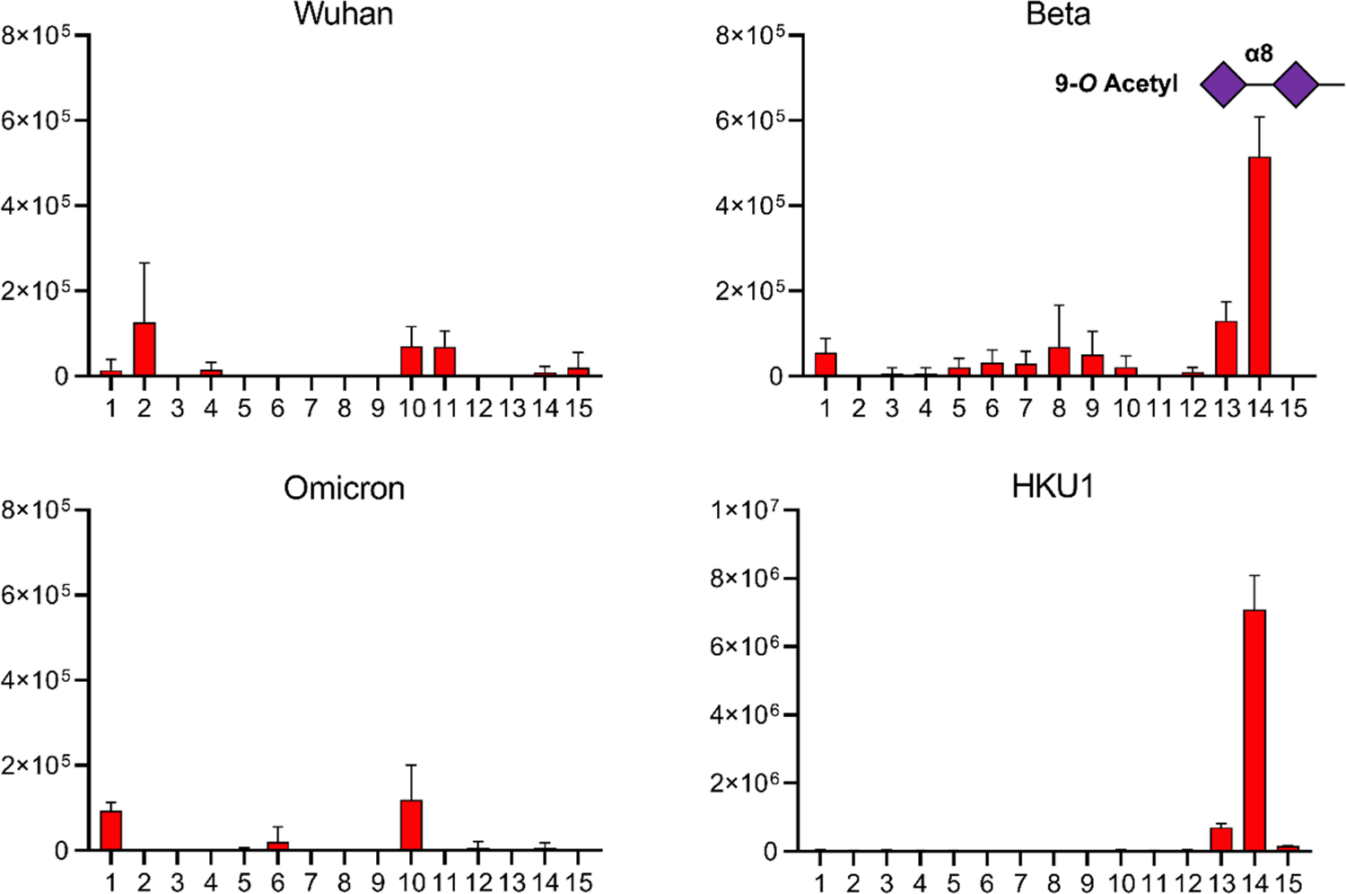
501Y.V2-1 NTD gains binding to 9-*O*-acetylated 2–8-linked disialic acid similarly to HKU1 NTD, with no binding being observed for SARS-CoV-2 Wuhan and omicron VoC.

Binding of 501Y.V2-1 NTD to Vero E6 cells and tissue slides and subsequent abrogation of binding by sialidase treatment instigated further characterization of receptor specificity. Glycan microarray technology was utilized to determine which sialylated structures can be bound. However, it must be noted that previous glycan microarrays^8,49−53^ were not able to fully characterize beta NTD glycan interactions, as no binding was observed. Since spike proteins of human coronaviruses (OC43 and HKU1) bind to 9-*O*-acetylated sialic acids, characterization of receptor specificity of 501Y.V2-1 NTD was, therefore, assessed using a glycan microarray with a collection of *O*-acetylated sialoglycans to determine comparable specificity. Glycan microarrays without acetylated sialoglycans did not display any binding interactions. The SARS-CoV-2 Wuhan strain, alpha, delta, and omicron variants did not display any responsiveness to *O*-acetylated sialic acids, whereas for the 501Y.V2-1 variant, NTD responsiveness was observed toward an α2–8-linked disialic acid structure containing 9-*O*-acetyl (#14) (Figures 4 and S3 and Table S2). This specificity was remarkably similar to HKU1, OC43, and influenza D (OK/D) HEF, which displayed a much broader *O*-acetylated sialic acid receptor binding capacity (Figure S3). Lower responsiveness was observed for 501Y.V2-1 NTD compared to that of HKU1, OC43, and OK/D, possibly related to the presence of hemagglutinin esterase activity, while SARS-CoV-2 lacks this activity and therefore requires a modest, reversible interaction.

### CaR-ESI-MS and Electrochemical Sensor Screening of 501Y.V2-1 NTD to Assess Receptor Specificity toward 9-*O*-Acetylated Disialosides

**Figure 5:**
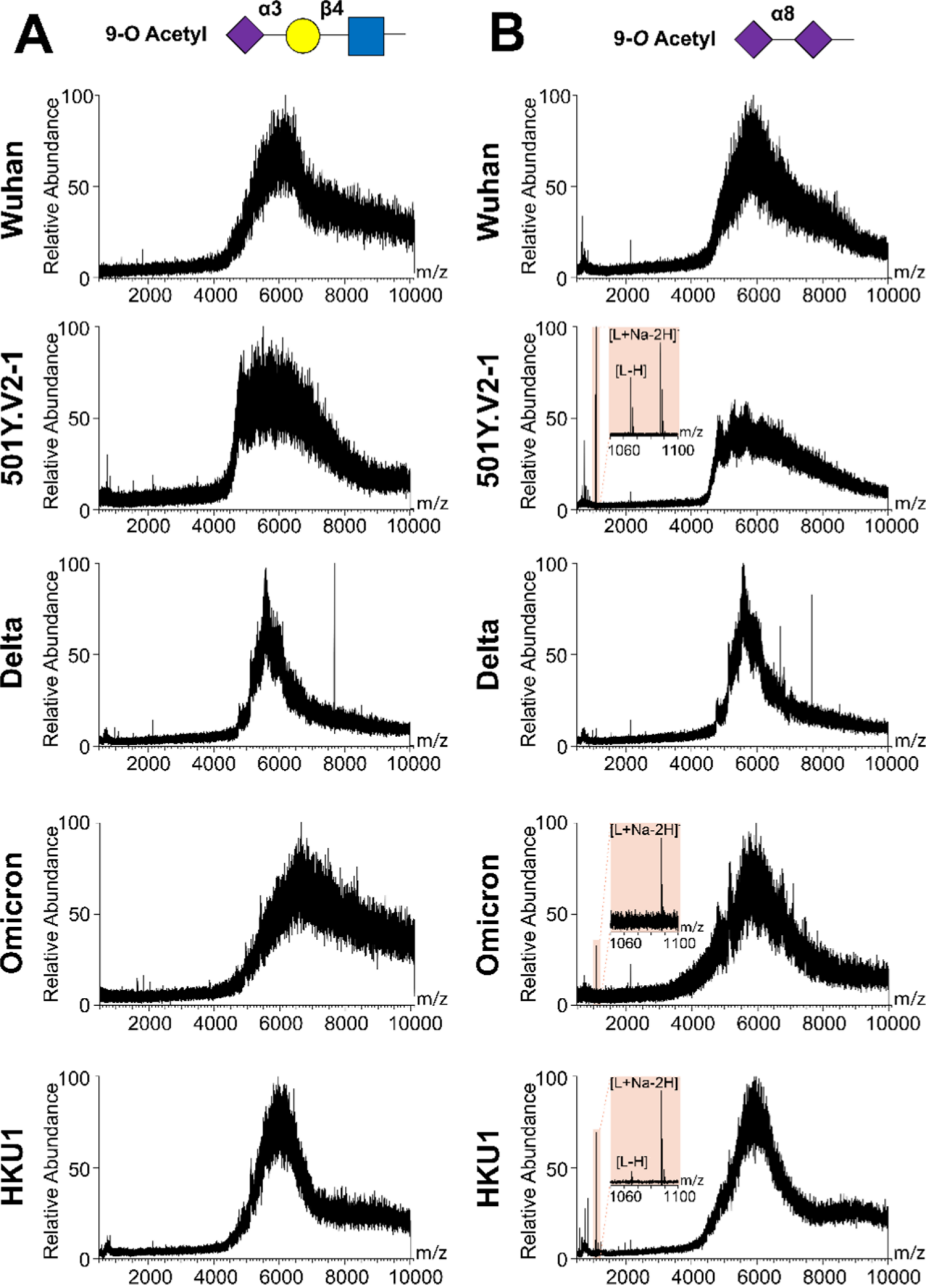
ESI-MS analysis confirms 9-*O*-acetylated sialic acid binding CaR-ESI-MS screening results obtained for aqueous ammonium acetate solutions (200 mM, pH 7.4, 25 °C). SARS-CoV-2 Wuhan, 501.V2-1, delta, omicron, and HKU1 NTDs in combination with 10 nM of 9-*O*-Ac 3-sialyllactosamine (A) or 9-*O*-Ac α2–8 disialic acid (B). Ions with *m*/*z* of 5000–7000 were subjected to HCD using a collision energy of 160 V. Screening with 9-*O*-acetyl α2–3-sialyllactosamine using five NTDs did not result in glycan release, while for 501Y.V2-1, omicron, and HKU1 NTD, glycan release was observed with 9-*O*-acetyl α2–8-linked disialic acid (highlighted region).

Further characterization of 9-*O*-acetylated disialoside responsiveness was performed using catch-and-release electrospray ionization mass spectrometry (CaR-ESI-MS) experiments to verify the specificity of 501Y.V2-1 NTD. CaR-ESI-MS is a semiquantitative highly sensitive screening technique that allows for label-free detection of weak glycan–lectin interactions. Identification of ligands is achieved after their release (as ions) from glycan–lectin complexes following collision activation in the gas phase.^8,54,55^ This approach was used to screen the specificity of SARS-CoV-2 Wuhan, 501Y.V2-1, delta, omicron, and HKU1 NTDs against 9-*O*-acetyl α2–8-linked disialic acid or 9-*O*-acetyl α2–3-sialyllactosamine, which did not display any responsiveness on the glycan microarray. For all screening experiments, aqueous solutions composed of the volatile ammonium acetate salt (200 mM, pH 7.4, 25 °C), NTD (10 μM), and 9-*O*-acetyl α2–8-linked disialic acid or 9-*O*-acetyl α2–3-sialyllactosamine (10 nM of each glycan) were used. Representative CaR-ESI mass spectra acquired in negative mode are shown in Figure 5. NTD–ligand complex ions with *m*/*z* values in the range of 5000–7000 were isolated for HCD. Over a range of collision energies (120–160 V), 9-*O*-acetyl α2–8-linked disialic acid was released, intact, as deprotonated (*m*/*z* 1065.45) or sodium adduct ions (*m*/*z* 1087.43) from the 501Y.V2-1 (highlighted region), omicron, and HKU1 NTD–ligand complexes. Notably, CaR-ESI-MS screening of five NTDs against 9-*O*-acetyl α2–3-sialyllactosamine produced no detectable released ligand signal, thus providing a blank experiment.

**Figure 6:**
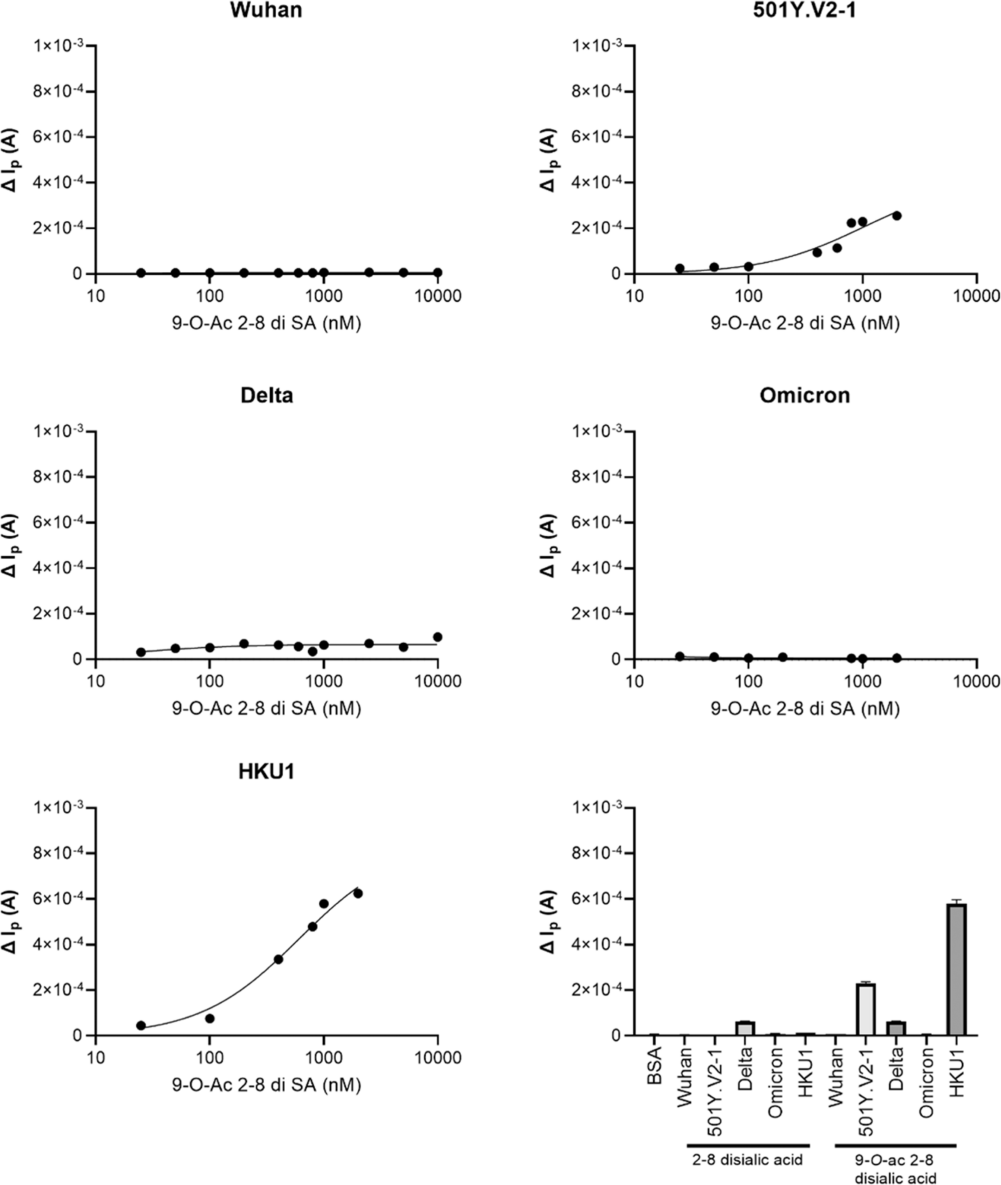
Graphene-based biosensor verifies 501Y.V2-1 NTD and 9-*O*-acetylated 2–8-linked disialic acid interaction. Screening of SARS-CoV-2 Wuhan, 501Y.V2-1, delta, omicron, and HKU1 NTD using graphene-based biosensor against 9-*O*-acetylated α2–8-linked disialic acid in a concentration-dependent manner, with positive correlation being observed for 501Y.V2-1 and HKU1 NTD. Use of nonacetylated α2–8-linked disialic acid did not result in glycan–protein interaction for SARS-CoV-2 Wuhan, 501Y.V2-1, delta, omicron, and HKU1 NTD, while signal is detected for beta and HKU1 NTD using 9-*O*-acetylated α2–8-linked disialic acid.

Further analysis was performed with a screen-printed electrode sensor using differential pulse voltammetry. This technique characterizes the oxidation in peak current as a function of the ferro/ferricyanide redox reaction that is influenced by the barriers created on the electrode surface (i.e., ligand to protein binding) by obstructing the diffusion of [Fe(CN)_6_]^3–/4–^.^56,57^ For the surface-coated SARS-CoV-2 Wuhan, delta, and omicron NTD, no signal was detected when using nonacetylated α2–8 disialic acid and 9-*O*-acetylated α2–8 disialic acid structures (Figure 6). Delta NTD occasionally displayed minimal background signal. The strongest binding signals were detected for HKU1 NTD to 9-*O*-acetyl α2–8-linked disialic acid followed by 501Y.V2-1 NTD, thus further verifying the glycan–lectin interaction of 501Y.V2-1 and HKU1 NTD to the 9-*O*-acetylated α2–8-linked disialic acid in a concentration-dependent manner. Binding of 501Y.V2-1 and HKU1 NTD was not detected toward nonacetylated α2–8 disialic acid (Figure 6).

### Identifying the Binding Epitope of 501Y.V2-1 NTD on the 9-*O*-Acetylated Ligand

**Figure 7:**
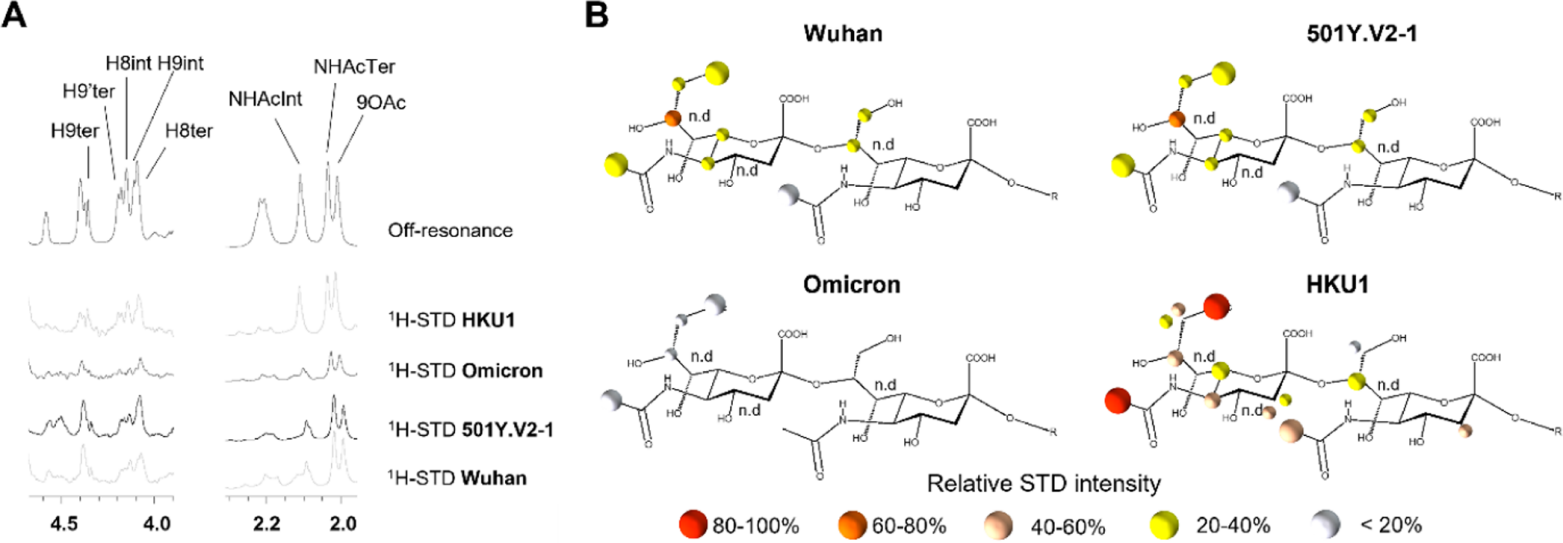
^1^H STD-NMR experiments for the interaction of the spike proteins of SARS-CoV-2 variants and HKU1 with the 9-*O*-acetyl α2–8-linked disialic acid. (A) Selected area of ^1^H STD-NMR spectra with aliphatic protein irradiation. (B) Ligand epitope mapping presented as relative STD intensities.

**Figure 8:**
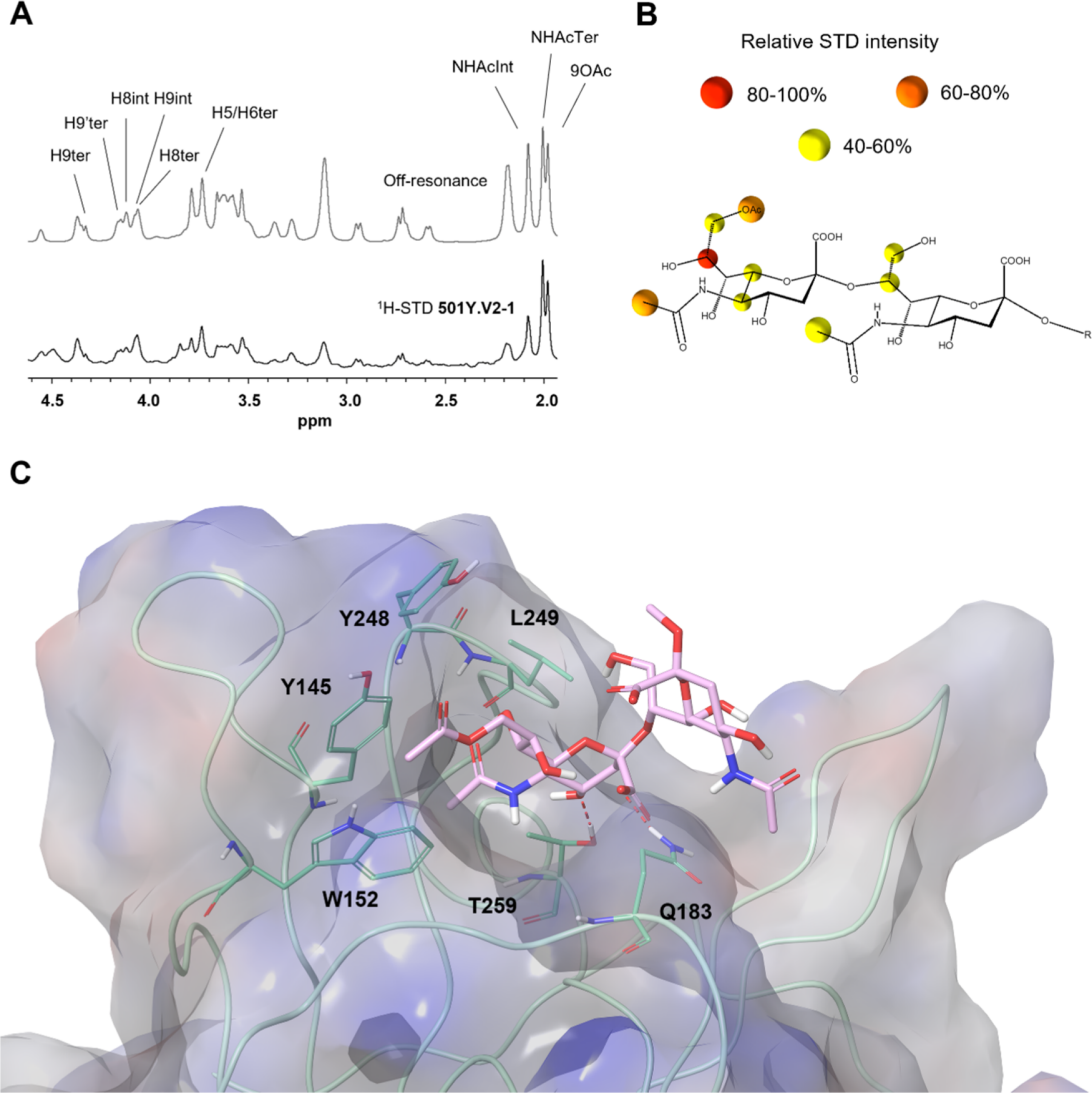
Interaction of the spike proteins of SARS-CoV-2 501Y.V2-1 with 9-*O*-acetyl α2–8-linked disialic acid. (A) ^1^H STD-NMR experiment. (B) Ligand epitope mapping presented as relative STD intensities. (C) Molecular model as derived by docking of the ligand into the sialic acid binding site. PDB code 7QUR.^23^

Glycan microarray, CaR-ESI-MS, and electrochemical analysis were elucidated to which structure 501Y.V2-1 NTD binds to. Next, STD-NMR was employed, which allows for the identification of the binding epitopes within the ligand to a target receptor.^58−60^ In STD-NMR, some protons within the protein are selectively irradiated with low power radiofrequency. Under spin diffusion conditions, the magnetization is quickly transferred to all of the protons of the receptor, which results in efficient protein saturation. If binding occurs, magnetization is transferred from the protein to the ligand protons. Importantly, not all protons of the ligand receive the same amount of saturation. Protons that are in closer proximity to the protein receive the strongest saturation, while the more distant will receive low saturation or none. Therefore, the resulting STD-NMR spectrum, which only contains the ligand protons that are affected by the protein saturation, not only detects binding or nonbinding but also informs about which part of the ligand is in closer contact with the protein. Thus, ^1^H STD-NMR experiments were performed to determine whether the SARS-CoV-2 Wuhan, 501Y.V2-1, omicron, and HKU1 NTDs bind the 9-*O*-acetyl α2–8-linked disialic acid and to define the corresponding ligand epitope (Figures 7 and 8). The results from this analysis showed that under these experimental conditions (in solution, using a relatively high ligand 0.8 mM concentration) SARS-CoV-2 Wuhan, 501Y.V2-1, and HKU1 NTD indeed recognize 9-*O*-acetyl α2–8-linked disialic acid as a ligand (Figure 7). For SARS-CoV-2 Wuhan and 501Y.V2-1, the main ligand epitope is the terminal sialic acid, with the strongest ^1^H STD-NMR signals arising from the 9-*O*-acetylated glycerol chain, indicating that this fragment is tightly interacting with these NTD proteins. Additional STD-NMR signals were detected from the methyl groups of the acetamide group at C-5 of both sialic acid moieties. Medium–weak STD-NMR signals were also observed from the H-5 and H-6 of the terminal sialic acid, while the protons at the C-3 provide STD-NMR signals neither in the terminal nor in the reducing end sialic acids. To complement these STD-NMR experimental-derived results, a putative complex of the NTD bound to 9-*O*-acetyl α2–8-linked disialic acid was built by using molecular modeling tools (Figure 8). According to the generated model, the carboxylate of the terminal sialic acid establishes a hydrogen bond interaction with the Q183 side chain, the acetyl moiety at the C-9 fits into a protein hydrophobic pocket defined by residues W152 and Y145, while the acetamide group at the C-5 is accommodated into a second pocket flanked by L249 and T259. Finally, the acetamide of the sialic acid at the reducing end also faces the protein surface, although no specific intermolecular interactions were found. The same analysis was performed for the omicron variant. Minimal ^1^H STD-NMR signals were detected for this variant in comparison to SARS-CoV-2 Wuhan, suggesting that the omicron NTD has lost its ability to efficiently recognize the 9-*O*-acetyl α2–8-linked disialic acid molecule as a ligand (Figure 7B). Finally, the NTD of HCoV-HKU1 was characterized, consistently, and the ^1^H STD-NMR spectrum of the corresponding complex with the 9-*O*-acetyl α2–8-linked disialic acid displayed the strongest STD intensities, with the main ligand epitope involving the acetyl moieties of the terminal sialic acid. Medium–strong STD-NMR signals were detected for the glycerol chain and for H-5 and H-3 axial of the terminal sialoside, as well as for the acetamide moiety and the H-3 equatorial of the reducing end sialoside. Medium–weak STD signals were also recorded for one of the H9, H-6, and H-3 equatorial of the terminal sialoside and for the H-8 of the internal residue. In all cases, while some of the STD-NMR signals could not be properly quantified due to ^1^H-NMR signal overlap, the NTD-9-*O*-acetyl α2–8-linked disialic acid interaction was unambiguously proven.

## Discussion

SARS-CoV-2 spike displays differential abilities to bind to glycans;^5,8^ for SARS-CoV-2 NTD, only a weakly binding glycan site is observed when using highly sensitive methods.^22,23^ In this report, we have characterized, with biochemical assays, the glycan binding properties of NTDs of SARS-CoV-2 VoCs. The 501Y.V2-1 NTD is the first VoC that displays a clear receptor binding capacity; the D215G mutation and conservation of amino acids at positions 242–244 appeared to be essential for sialic acid-dependent binding. Glycan microarray, CaR-ESI-MS, STD-NMR, and electrochemical analysis validated 9-*O*-acetylated α2–8 disialic acid binding specificity of the 501Y.V2-1 NTD. The 501Y.V2-1 variant shows strong interaction with the 9-*O*-acetyl and NHAc-5 arm NTD of the ligand, similarly to HKU1, indicating a convergent evolution of coronaviruses toward host adaptation and 9-*O*-acetylated sialoside recognition using their NTD.^7,11,22,23,27,61−64^ Omicron NTD interaction with 9-*O*-acetylated 2–8 disialic acid was minimally detected with CaR-ESI-MS; weak binding was observed with STD-NMR and confocal imaging, whereas binding was not observed with a glycan array and a graphene-based biosensor. Low-affinity interaction with acetylated structures may be possible for other VoCs; however, this could not be validated.

Initial virus–receptor interaction may vary in affinity and avidity, whereby engagement first occurs with a low-affinity/high-avidity interaction (glycan binding) and then potentially followed by a stronger secondary receptor interaction (protein binding).^65^ Many viruses utilize reversible low-affinity interaction through binding with (sialylated) glycans to mediate viral surfing.^3,29,66^ Coronaviruses utilize their NTD for sialic acid binding and promote infection.^7,28,37,38,64,67^ So far, only MERS-CoV has been identified as using a two-step attachment mechanism that contributes toward tropism by utilizing its S1-NTD to interact with α2–3 sialic acid structures (viral surfing).^68^ Even though a difference in the sequence is observed among HCoV-HKU1, MERS-CoV, and SARS-CoV-2, these proteins share a high protein folding similarity and structural overlap whereby the sialic acid binding site appears to be conserved.^7,69−71^ In silico structural and molecular studies elaborate that the flat and nonsunken NTD surface of SARS-CoV-2 should allow for sialic acid interactions, enabling viral surfing and dual-receptor functionality of the S1 spike.^72−74^ S1 spike interaction with sialic acids has been further shown by four independent studies; however, the contribution of S1-NTD toward sialoside recognition and dual-receptor functionality has not been verified.^8,10,11,75^

STD-NMR experiments^23^ have identified a clear “end-on” interaction of S1-NTD with α2–3- or α2–6-linked sialic acids, describing a cryptic sialic acid binding domain. A cryo-EM-derived map of the S1-NTD has proposed H69, Y145, and S247 as the sialic acid-interacting amino acids.^23^ The amino acid at position Y145 interacts with the glycerol moiety (C7–C9) of the terminal sialic acid with a hydrogen bond and the S247 amino acid engaging the NHAc-5 arm, which is also supported by computational modeling described in our report. Numerous mutations in the sialoside binding site of VoCs appear to remove essential interacting residues (H69, V70, Y145: alpha and omicron) or perturb the β-sheet structure that forms the binding pocket (L242–244del: beta).

Interestingly, when using α2–3-, α2–6-, and α2–8-linked *O*-acetylated sialic acids in our glycan array, binding was only observed for 9-*O*-acetyl α2–8-linked sialic acids when using 501Y.V2-1 NTD. 9-*O*-Acetylated sialylated structures are variably displayed and can be found in the submucosal glands and alveolar pneumocytes of humans, hence functioning as an initial binding point for 501Y.V2-1 NTD.^30,33,35^ Previously, the inhibition of S1 spike binding was attained with Neu5Ac monosaccharides,^23,75^ α2–3-linked,^23^ (multivalent) α2–6-linked,^10,23^ and (multivalent) 9-*O*-acetylated sialic acid,^11^ which raises questions regarding the specificity of SARS-CoV-2 NTD toward these structures and whether sufficient receptor binding can be mediated to support a dual-receptor function. Thus, the current reported knowledge indicates that sialic acid binding and multivalency are crucial in infection and tropism while the exact glycan partner can vary between coronavirus strains.^76^

The lectin function for 501Y.V2-1 VoC is a clear convergent evolutionary path of adaptation to 9-*O*-acetylated sialoside recognition by the NTD, similarly to β-CoV HKU1 and OC43.^7,11,22,23,27,61−64^ However, ablation in later beta/501Y.V2 variants induced by the L242–244deletion^45,46^ and in other VoC (alpha, delta, and omicron) raises the question of why this increased binding capacity toward sialic acids is lost. Influenza, HKU1, and OC43 possess a receptor-destroying enzyme (neuraminidase and hemagglutinin esterase) that results in the “release” of virions from host cells.^77^ OC43 without a hemagglutinin esterase (HE) activity and influenza viruses without neuraminidase activity have poor viral dissemination by preventing release from infected host cells.^78,79^ This may explain why in the dominant beta/501Y.V2-3 variant enhanced sialic acid binding was abrogated. As SARS-CoV-2 does not possess HE on its viral membrane,^80^ a balance is needed between the ability to bind host cells for initial infection (tropism) and dissemination in the host (viral release). Similar to SARS-CoV-2, the MERS-CoV strain does not possess HE activity, although a dual-receptor functionality has been observed through α2–3-linked sialic acid interaction of the NTD. Interestingly, this interaction of MERS-CoV NTD with sialic acids appears to be extremely weak compared to HKU1 and OC43 NTD. Multimerization with a nanoparticle is required for MERS–NTD to observe sialic acid binding,^68^ while HKU1 and OC43 NTD have a strong affinity toward sialic acids,^7,28^ suggesting that MERS-CoV retains a fine balance between sialic acid binding and subsequent release from infected cells using its NTD.

In summary, the observation that 501Y.V2-1 NTD has gained a lectin function supports the dual-receptor notion of S1 spike, similar to that of MERS-CoV, showing that SARS-CoV-2 might probe evolutionary space to allow for alternative or additional receptor binding. Subsequent ablation in VoC raises questions regarding the convergent evolutionary path of glycan binding proteins to recognize (acetylated) sialylated glycan structures and the fine balance it requires for infection and/or transmission.

## Materials and Methods

### SARS-COV-2 NTD Expression Plasmid Generation

Recombinant SARS-CoV-2 spike protein NTD (GenBank: MN908947.3; AA319-541), OC43 NTD (GenBank: L14643.1; AA15-302), and HKU1 NTD (GenBank: DQ339101; AA14-294) were cloned using Gibson assembly from cDNAs encoding codon-optimized open reading frames of full-length SARS-CoV-2 spike,^81^ as previously described.^43^ Influenza D (OK/D) HEF construct has been described previously.^82^ The pCD5 expression vector was adapted so that after the signal sequence, the SARS-COV-2, OC43, and HKU1 NTD-encoding cDNAs are cloned in frame with a GCN4 trimerization motif (KQIEDKIEEIESKQKKIENEIARIKK), a TEV cleavage site, fluorescent reporter open reading frame,^83,84^ and the Strep-Tag II (WSHPQFEKGGGSGGGSWSHPQFEK); IBA, Germany.

### Protein Expression and Purification

pCD5-SARS-COV-2 NTD- + GCN4 - fluorescent probe expression vectors were transfected into HEK 293T with polyethyleneimine I (PEI) in a 1:8 ratio (μg DNA/μg PEI) as previously described.^85^ The transfection mix was replaced after 6 h by 293 SFM II suspension medium (Invitrogen, 11686029, supplemented with glucose 2.0 g/L, sodium bicarbonate 3.6 g/L, primatone 3.0 g/L (Kerry), 1% glutaMAX (Gibco), 1.5% DMSO and 2 mM valproic acid). Culture supernatants were harvested 5 days post-transfection. The SARS-COV-2 NTD expression was analyzed with SDS-PAGE followed by Western blot on the PVDF membrane (Biorad) using α-strep-tag mouse antibodies 1:3000 (IBA Life Sciences). Subsequently, SARS-COV-2 NTD proteins were purified with Sepharose Strep-Tactin beads (IBA Life Sciences) as previously described.^85^

### Immunofluorescent Cell Staining

Vero E6 and HEK 293T, grown on coverslips in a 24-well plate, were analyzed by immunofluorescent staining. Cells were fixed with 4% paraformaldehyde in PBS for 25 min at RT after which permeabilization was performed using 0.1% Triton in PBS. Subsequently, the coronavirus spike proteins were applied at 50 μg/mL and precomplexed with primary StrepMAB-Classic-HRP (IBA) and secondary Alexa-fluor555 goat antimouse (Invitrogen) at a 4:2:1 molar ratio. SNA (B-1305-2, Vectorlabs) or ECA (B-1145-5, Vectorlabs) was applied at 10 μg/mL precomplexed with 2.5 μg/mL of streptavidin488 (S11223, Thermo Fisher) for 1 h at RT. VCNA treatment was performed with neuraminidase (P0722L, New England Biolabs) in 10 mM potassium acetate pH 4.2 and 0.01% Triton X-100. 3FNeu5Ac (#5760, Biotechne) was dissolved in DMSO and used in a final concentration of 300 μm, and incubation was performed for 72 h for sialic acid depletion of surface glycans. DAPI (Invitrogen) was used as nuclear staining. Samples were imaged on a Leica DMi8 confocal microscope equipped with a 10× HC PL Apo CS2 objective (NA 0.40). Excitation was achieved with a Diode 405 or white light for excitation of Alexa555, and a pulsed white laser (80 MHz) was used at 549 nm; emissions were obtained in the range of 594–627 nm. Laser powers were 10–20% with a gain of a maximum of 200. LAS Application Suite X was used as well as ImageJ for the addition of the scale bars.

### Glycan Array

Acetylated structures were printed on glass slides as previously described.^28^ The glycan microarray was utilized as described previously for NTD proteins.^86^ Precomplexation was performed with NTD proteins using StrepMAB-Classic-HRP (IBA) and goat antimouse-Alexa555 antibodies in a 4:2:1 molar ratio, respectively, in 50 μL of phosphate-buffered saline (PBS) with 0.1% of Tween 20. Samples were incubated on ice for 15 min, followed by incubation on the array for 90 min in a humidity chamber. Slides were rinsed with Tween 20, PBS, and deionized water, followed by centrifugation and scanning as described previously.^86^ Data processing was performed using six replicates; the lowest and highest replicates were removed, and subsequent mean and standard deviation were calculated using the remaining four replicates.

### ESI-MS

#### Proteins and Glycans

Protein stock solutions were dialyzed against 200 mM of aqueous ammonium acetate pH 7.4 using an Amicon 0.5 mL of microconcentrator with an MW cutoff of 10 kDa (EMD Millipore, Billerica, MA) and stored at 4 °C until needed. The concentration of each protein stock solution was estimated by UV absorption at 280 nM.

Stock solutions of each glycan were prepared by dissolving a known mass in 100 mM ammonium bicarbonate (pH 7.4) with ultrafiltered water (Milli-Q Millipore, MA) to achieve a final concentration of ∼1 mM. All stock solutions were stored at −20 °C until use.

#### Mass Spectrometry

The CaR-ESI-MS experiments were performed in negative mode using a Q Exactive Ultra-High Mass Range Orbitrap mass spectrometer (Thermo Fisher Scientific). The mass spectrometer was equipped with a modified nanoflow ESI (nanoESI) source. NanoESI tips with an outer diameter (o.d.) of ∼5 μm were pulled from borosilicate glass (1.0 mm o.d., 0.78 mm inner diameter) with a P-1000 micropipette puller (Sutter Instruments). A platinum wire was inserted into the nanoESI tip, making contact with the sample solution. A voltage of approximately −1 kV was applied to the platinum wire.

The capillary temperature was 150 °C, and the S-lens RF level was 100; an automatic gain control target of 1 × 10^6^ and a maximum injection time of 200 ms were used. The resolving power was set to 25,000. HCD spectra were acquired using collision energies ranging from 10 to 300 V. Argon was used for collision-induced dissociation (CID) at a trap ion guide pressure of 1.42 × 10^–2^ mbar. Data acquisition and preprocessing were performed using Xcalibur version 4.1.

### Graphene-Based Biosensor

#### Fabrication of the Electrochemical Graphene-Based Biosensor

A 10 μL mixture of 1 μM MUA and 10 μM DTT were dropped onto the AuNPs/G/GCE surface of the electrode and placed in a refrigerator (4 °C) for 14 h to obtain the MUA/AuNPs/GGCE. The as-prepared electrode was activated in 100 μL of a freshly prepared solution containing 2 g/L of EDC and 0.5 g/L of NHS for 30 min to activate the carboxylic groups on MUA. Then, the activated electrode was immersed in 100 μL of lectin (10 μM) or spike protein (100 nM) solution for 1 h. The spike protein/MUA/AuNPs/G/GCE was immersed in 100 μL of 1% BSA for 30 min to inhibit nonspecific interactions, and then the electrode was rinsed thoroughly to remove any adsorbed components. The spike protein/AuNPs/G/GCE was stored at 4 °C in PBS (pH 7.4).

#### Electrochemical Measurements

Electrochemical measurements were performed in 100 μL of analyte, which includes 10 mM PBS containing 25 mM [Fe(CN)_6_]^3–/4–^ and 0.2 M KCl. Cyclic voltammetry (CV) was used to monitor the fabrication process of the biosensor. All of the CV voltammograms were recorded from −0.2 to 0.8 V (vs Ag/AgCl) at a scan rate of 0.05 V/s. Differential pulse voltammetry (DPV) was used as the validation method. All DPV voltammograms were recorded from −0.2 to 0.5 V (vs Ag/AgCl) at a modulation time of 0.05 s, a modulation amplitude of 0.1 V, and an interval time of 0.5 s. All electrochemical experiments were performed at room temperature (25 ± 1 °C). Glycan ligands were added to reach a final concentration of 10,000 nM.

### STD-NMR

^1^H STD-NMR experiments were acquired on a Bruker 800 MHz spectrometer with a cryoprobe (Bruker, Billerica, MA) at 298 K. Proteins was buffer-exchanged by 20 mM phosphate buffer (pD 7.5) containing 150 mM NaCl and 0.05% sodium azide in D_2_O. All of the proteins were concentrated to a final concentration of 8 μM. The glycan ligands were then added to reach a final concentration of 800 μM, which lead to a protein/ligand ratio of 1:100. ^1^H STD-NMR spectra were acquired by using a standard Bruker STD sequence (stddiffesgp.3) with 1152 scans in a matrix with 64 K data points in a spectral window of 12335.5 Hz centered at 2818 Hz. An excitation sculpting module with gradients was used to suppress the water proton signals and a protein suppression spin lock filter of 40 ms. Protein’s resonance selective saturation was reached by irradiating at −0.2 ppm (aliphatic residues) using a series of 40 Eburp2.1000-shaped 90° pulses (50 ms) for a total saturation time of 2 s and a relaxation delay of 3 s. For the reference spectrum, an irradiation frequency of 100 ppm was used. Control STD-NMR experiments were performed for both the only ligands and apo proteins using the same STD experimental setup. Spectral analysis determined the percentages of STD intensities as estimated by comparing the intensity of the signals in the STD spectrum with the signal intensities of the off-resonance spectrum. The STD intensities of the ligands in the absence of the protein were subtracted. The STD-derived epitope maps are represented as the relative percentages of each ligand signal with respect to the highest one. Resonances are labeled as n.d. (not determined) when the ^1^H-NMR signal degeneration hampers rigorous quantitative analysis.

## Supporting Information Available

Figures S1 and S2 contain confocal images of NTD and lectin stains on Vero E6 cells to verify binding under different conditions; Figure S3 contains glycan array data of NTDs and displays receptor recognition; and Tables S1 and S2 contain structures recognized by different coronaviruses and glycan structures presented on the *O*-acetylated glycan array (PDF)
