# Supplementary material for: SARS-CoV-2 Spike N-Terminal Domain Engages 9-O-Acetylated α2–8-Linked Sialic Acids
Source: ACS Chem Biol. 2023 Apr 27;18(5):1180–91. doi: 10.1021/acschembio.3c00066 (PMC10178783; doi:10.1021/acschembio.3c00066)
Supplement: Supplementary file 1 — cb3c00066_si_001.pdf [file cb3c00066_si_001.pdf]

Supporting Information

SARS-CoV-2 Spike N-Terminal Domain Engages 9-*O*-Acetylated α2-8-Linked Sialic Acids

Ilhan Tomris[1#], Luca Unione[2,3#], Linh Nguyen[4#], Pouya Zaree[1#], Kim M. Bouwman[1#], Lin Liu[5], Zeshi Li[1], Jelle A. Fok[1], María Ríos Carrasco[1], Roosmarijn van der Woude[1], Anne L.M. Kimpel[1], Mirte W. Linthorst[1], Sinan E. Kilavuzoglu[1], Enrico C.J.M Verpalen[1], Tom G. Caniels[6,7], Rogier W. Sanders[6,7,8], Balthasar A. Heesters[1], Roland J. Pieters[1], Jesús Jiménez-Barbero[2,3,9,10], John S. Klassen[4], Geert-Jan Boons[1,5] and Robert P. de Vries[1*]

[1] Department of Chemical Biology & Drug Discovery, Utrecht Institute for Pharmaceutical Sciences, Utrecht University, 3584 CG Utrecht, The Netherlands.

[2] CICbioGUNE, Basque Research & Technology Alliance (BRTA), Bizkaia Technology Park, Building 800, 48160 Derio, Bizkaia, Spain.

[3] Ikerbasque, Basque Foundation for Science, Maria Diaz de Haro 3, 48013 Bilbao, Bizkaia, Spain.

[4] Department of Chemistry, University of Alberta, 11227 Saskatchewan Dr, Edmonton T6G 2G2, Canada.

[5] Complex Carbohydrate Research Center, University of Georgia, 315 Riverbend Rd Athens, Georgia 30602, USA.

[6] Department of Medical Microbiology, Amsterdam UMC, University of Amsterdam, 1081 HZ Amsterdam, the Netherlands.

[7] Amsterdam Institute for Infection and Immunity, Infectious Diseases, 1081 HZ Amsterdam, the Netherlands.

[8] Department of Microbiology and Immunology, Weill Medical Center of Cornell University, 1300 York Ave, New York, NY 10065, USA.

[9] Department of Organic Chemistry, II Faculty of Science and Technology University of the Basque Country, EHU-UPV, 48940 Leioa, Spain.

[10] Centro de Investigación Biomédica En Red de Enfermedades Respiratorias,

Av. Monforte de Lemos, 3-5. Pabellón 11. Planta 0 28029 Madrid, Spain.

[#] These authors contributed equally

[*] for correspondence: r.vries@uu.nl

Figure S1: Confocal Vero E6 staining

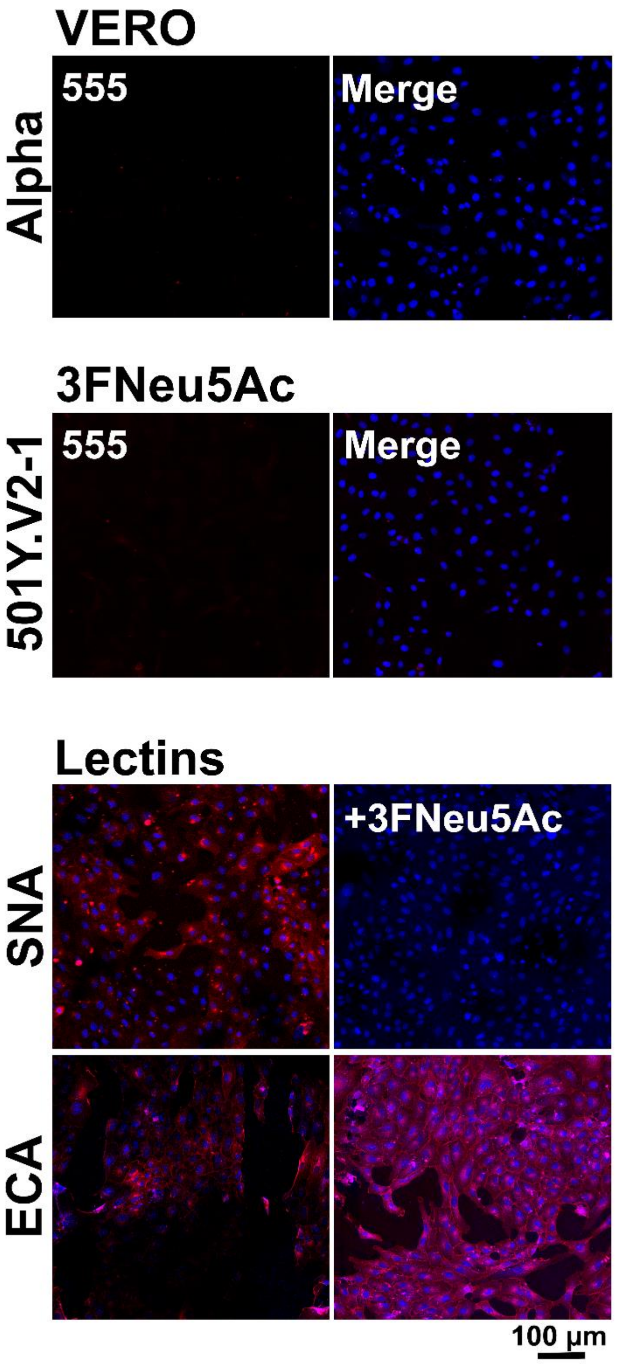

Figure S1: Confocal imaging of Alpha VoC NTD did not display any cell-binding properties. 3FNeu5Ac treatment of Vero cells abrogates binding of 501Y.V2-1. SNA and ECA lectin utilized to verify 3FNeu5Ac inhibition of sialic acid, SNA binding is abrogated after treatment and ECA binding is enhanced.

Figure S2: Confocal HEK293T and Vero E6 staining

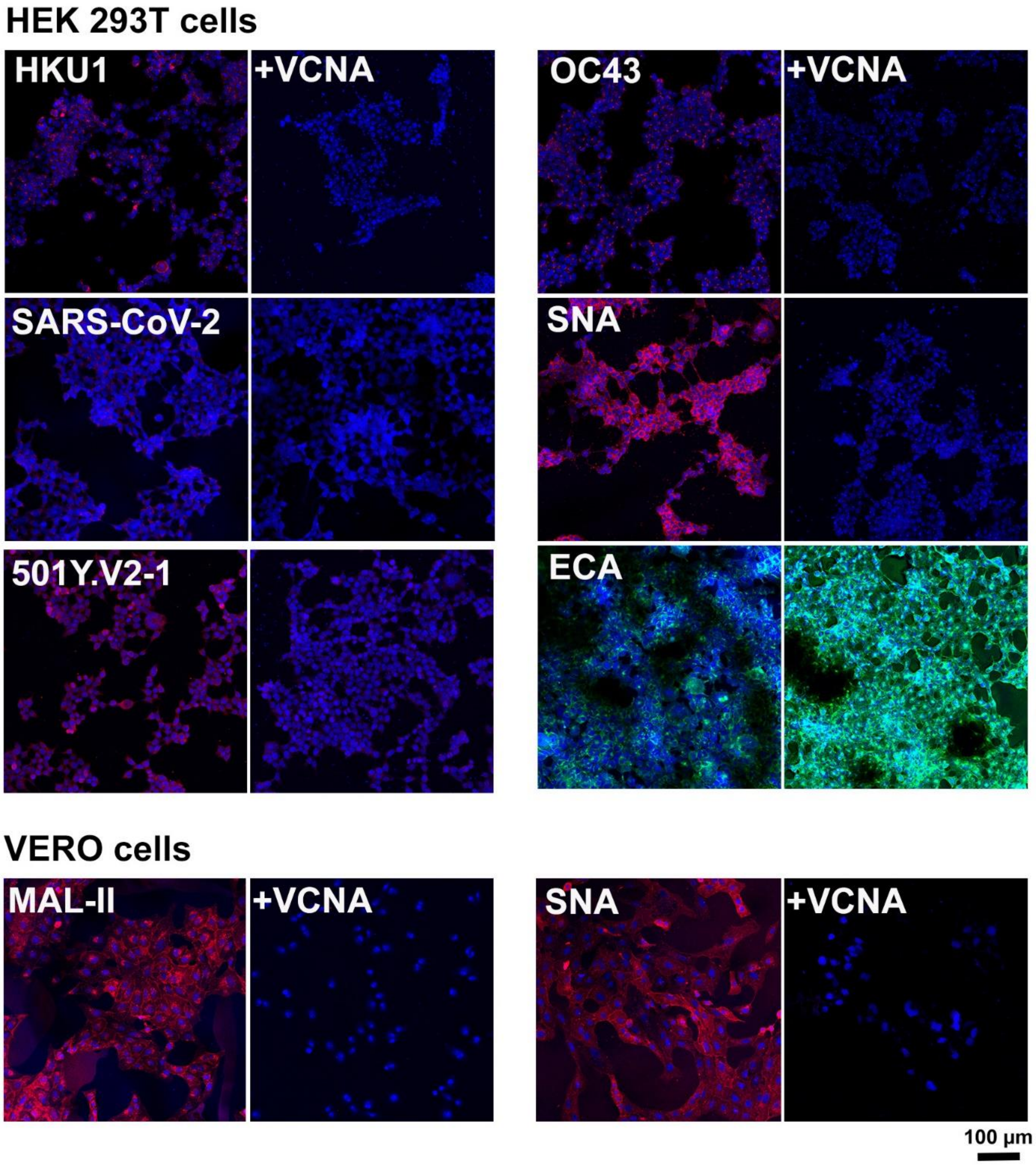

Figure S2: Sialic acid-dependent binding of HKU1, SARS-CoV-2 Wuhan, 501Y.V2-1 and OC43 NTD with SNA and ECA on HEK 293T cells with no ACE2 expression. Binding is abrogated after Vibrio cholerae neuraminidase (VCNA) treatment for all lectins except for ECA, which preferentially recognizes non-sialylated structures.

Figure S3: Glycan array

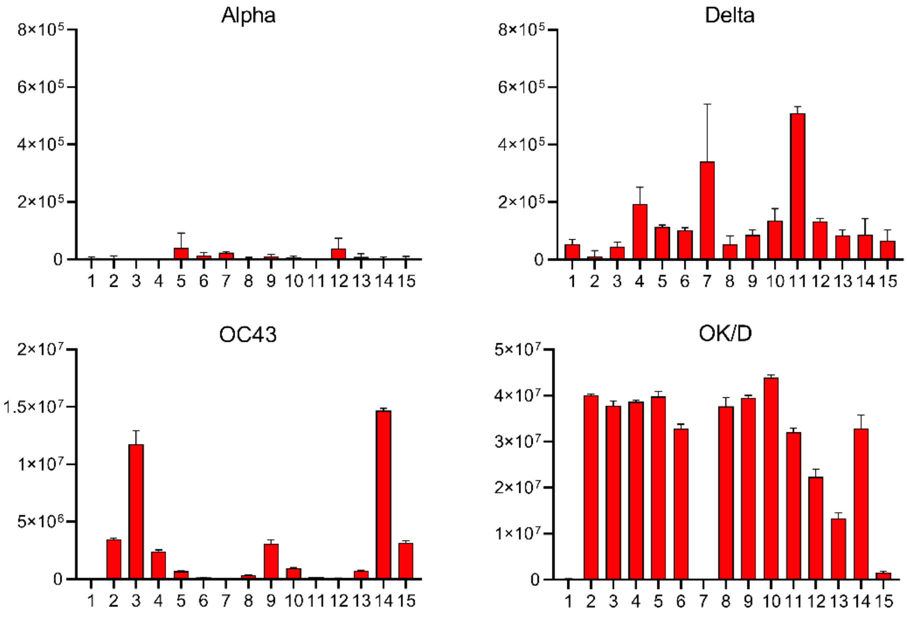

Figure S3: O-acetyl glycan array analysis did not elucidate binding capacity to acetylated structures using Alpha or Delta VoC NTDs, with only background signal being observed. OC43 and OK/D show specificity towards acetylated structures.

Table S1.

Virus	Target receptor	Reference
IBV	Neu5Acα2,3Galβ1,3GlcNAc	[1]
TCoV/GfCoV/QCoV	Galβ1,4GlcNAcβ1,3Galβ1,4GlcNAc	[2]
HKU1/OC43/BCoV	Neu5,9Ac$_2$	[3, 4]
MERS	Neu5Acα2,3Gal	[5]

Table S2.

Compound	IUPAC	Structure
1	Neu4,5Ac$_2$-α2,3-Gal-β1-4-GlcNAc	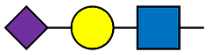
2	Neu5,7Ac$_2$-α2,3-Gal-β1-4-GlcNAc	
3	Neu5,9Ac$_2$-α2,3-Gal-β1-4-GlcNAc	
4	Neu4,5,9Ac$_3$-α2,3-Gal-β1-4-GlcNAc	
5	Neu5,7,9Ac$_3$-α2,3-Gal-β1-4-GlcNAc	
6	Neu4,5,7,9Ac$_4$-α2,3-Gal-β1-4-GlcNAc	
7	Neu4,5Ac$_2$-α2,6-Gal-β1-4-GlcNAc	
8	Neu5,7Ac$_2$-α2,6-Gal-β1-4-GlcNAc	
9	Neu5,9Ac$_2$-α2,6-Gal-β1-4-GlcNAc	
10	Neu4,5,9Ac$_3$-α2,6-Gal-β1-4-GlcNAc	
11	Neu5,7,9Ac$_3$-α2,6-Gal-β1-4-GlcNAc	
12	Neu4,5,7,9Ac$_4$-α2,6-Gal-β1-4-GlcNAc	
13	Neu5,7Ac$_2$-α2,8-Neu5Ac	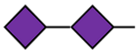
14	Neu5,9Ac$_2$-α2,8-Neu5Ac	
15	Neu5,7,9Ac$_3$-α2,8-Neu5Ac	

References

1. Wickramasinghe, I.N., et al., *Binding of avian coronavirus spike proteins to host factors reflects virus tropism and pathogenicity.* J Virol, 2011. **85**(17): p. 8903-12.
2. Ambepitiya Wickramasinghe, I.N., et al., *Novel Receptor Specificity of Avian Gammacoronaviruses That Cause Enteritis.* J Virol, 2015. **89**(17): p. 8783-92.
3. Hulswit, R.J.G., et al., *Human coronaviruses OC43 and HKU1 bind to 9-O-acetylated sialic acids via a conserved receptor-binding site in spike protein domain A.* Proc Natl Acad Sci U S A, 2019. **116**(7): p. 2681-2690.
4. Peng, G., et al., *Crystal structure of bovine coronavirus spike protein lectin domain.* J Biol Chem, 2012. **287**(50): p. 41931-8.
5. Li, W., et al., *Identification of sialic acid-binding function for the Middle East respiratory syndrome coronavirus spike glycoprotein.* Proc Natl Acad Sci U S A, 2017. **114**(40): p. E8508-E8517.
